# Antibiotic Resistance Patterns in Pancreatic Surgery: A Tailor-Made Antibiotic Prophylaxis in a Secondary Hospital

**DOI:** 10.7759/cureus.102254

**Published:** 2026-01-25

**Authors:** Catarina Guimaraes, Daniela Tavares, Mara Nunes, Tatiana Moreira Marques, Rita Peixoto, Pedro Soares-Moreira, Gil Faria

**Affiliations:** 1 General Surgery Department, Unidade Local de Saúde de Matosinhos, Matosinhos, PRT

**Keywords:** antibiotic prophylaxis, bacteriobilia, biliary drainage, pancreatic fistula, pancreatoduodenectomy

## Abstract

Background/objectives: The pancreatoduodenectomy is a high-risk surgery, and its high morbidity rate is mostly related to postoperative infectious complications. Positive bile cultures are associated with both increased frequency and severity of pancreatic fistulas; this is more common in previously drained or infected bile ducts. The currently used antibiotic regimens for surgical prophylaxis appear insufficient to cover the microorganisms identified in bile cultures of patients previously submitted to biliary drainage. This study aims primarily to compare the rates of positive bile cultures and antibiotic resistance between patients with and without preoperative biliary drainage.​ And, as a secondary aim, to evaluate whether these positive cultures are associated with an increased risk of postoperative pancreatic fistulas (POPF).

Methods: We conducted a retrospective single-center study of patients undergoing pancreatoduodenectomy to compare intraoperative bile samples from patients with prior bile drainage with those without prior bile drainage between 2015 and 2022. The main aim was to compare the percentage of positive bile cultures, as well as describe the microbiological patterns and the respective antibiotic susceptibility. The secondary aim was to compare postoperative complications, such as pancreatic fistula.

Results: During the study period, 69 patients underwent pancreatoduodenectomy, and 36 patients met the inclusion criteria. Of the 36 patients, 22 had been previously submitted to bile drainage, and 14 went straight to surgery. The mean age of the patients was 72.7 years, with 22 (61.1%) patients being male. The leading surgical indication was pancreatic adenocarcinoma. The presence of positive bile cultures was significantly higher in patients with previously performed bile drainage, with 20 (90.9%) having positive cultures; 16 (80%) of these patients presented with polymicrobial growth. Notably, *Klebsiella pneumoniae* and *Enterococcus faecium* were the most frequently isolated Gram-negative and Gram-positive bacteria, respectively. A significant proportion of bacteria exhibited resistance to the common surgical prophylaxis, with 17 (85%) of the instrumented group resistant to cefuroxime. Complications included an 11 (50%) rate of POPF in the drainage group and a 3 (21.4%) rate in the no drainage group, with no difference between the two groups.

Conclusions: In conclusion, our study shows that the frequency of positive bile cultures is much higher in patients previously submitted to biliary drainage, although this did not increase the risk of pancreatic fistula. After subgroup analyses, we concluded that the current standard antibiotic prophylaxis at our institution is inadequate to cover the microbiologic profile observed in our local patient cohort.

## Introduction

Pancreatoduodenectomy is a high-risk procedure, with a mortality rate of approximately 2-5% and a morbidity rate reaching up to 40% [1,2]. In most cases, the high morbidity rate is related to infectious complications like surgical site infections (SSIs). Some known risk factors for the existence of SSI are previous biliary drainage, previous positive bile cultures, and the presence of pancreatic fistula [3-5]. Most patients will present with organ/space infections, with a high percentage being related to a postoperative pancreatic fistula (POPF) [2,6]. Patients previously submitted to bile drainage have a higher percentage of bacteriobilia, which is associated with both higher frequency and severity of pancreatic fistulas [4]. This is common in patients with periampullary malignancies who develop obstructive jaundice, often requiring preoperative biliary drainage [7]. Although the use of broad-spectrum antibiotics appears to be associated with a reduction in complications and the formation of pancreatic fistula, the common antibiotic regimens used in surgical prophylaxis appear insufficient to cover the microorganisms identified in bile cultures [6,8-10].

The main aim of the current study is to compare two groups of patients who underwent pancreatoduodenectomy, dividing them into those previously submitted to biliary drainage and those who were not. In a subgroup analysis, the aim is to evaluate the antibiotic susceptibility and resistance patterns of the microorganisms in bile cultures of each group. As a secondary aim, a description of the number and severity of POPF was made.

## Materials and methods

**Figure 1:**
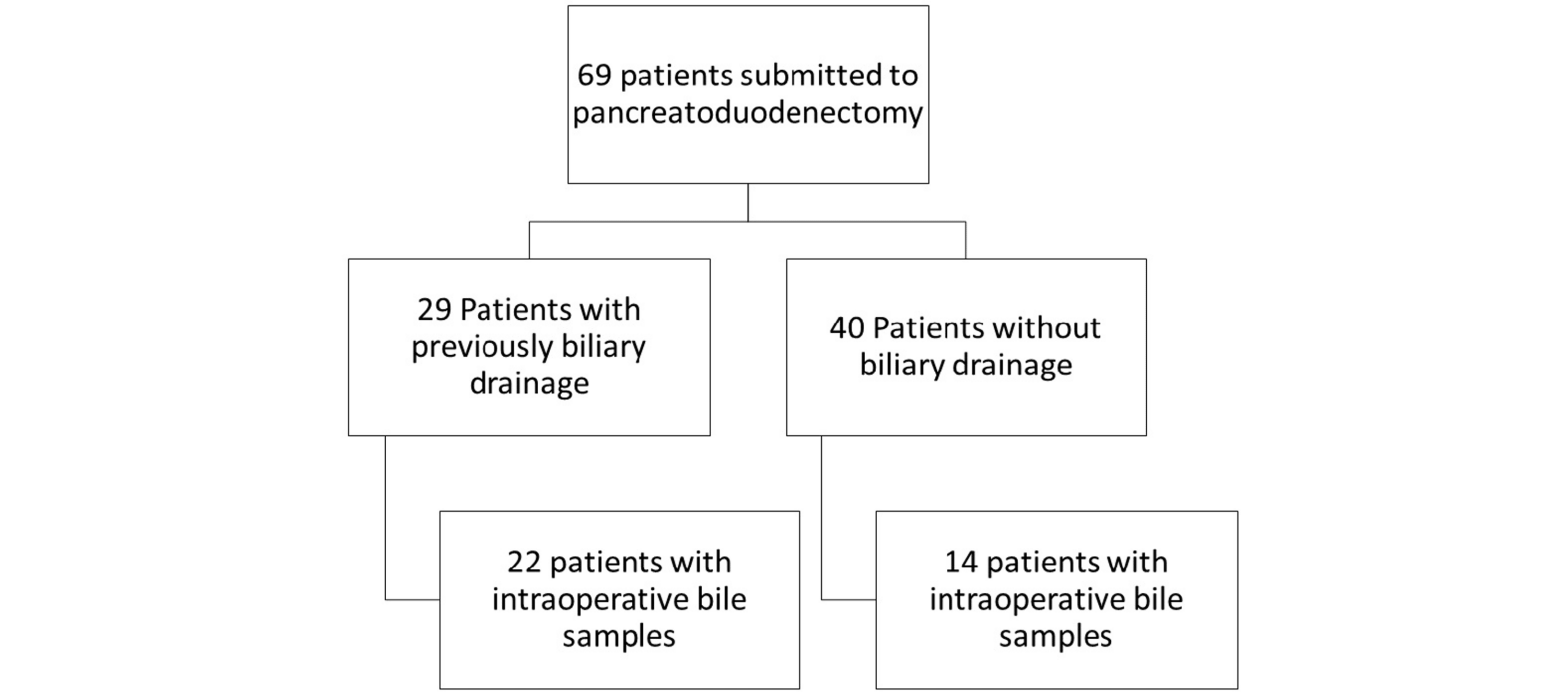
Patient selection Patients submitted to pancreatoduodenectomy during the established period. After applying the exclusion criteria, 22 patients remained in the drainage group, and 14 patients remained in the group without previous drainage. The last row shows the patients selected for the study.

We started by selecting the patients submitted to pancreatoduodenectomy during the period from March 2015 to March 2022 for periampullary malignancies; 69 patients underwent surgery during this period. We divided the patients into two groups: those with prior biliary drainage (Group 1) and those without biliary drainage (Group 2). Since the focus was to evaluate the prevalence of bile colonization in patients previously submitted to biliary drainage, all patients without bile samples obtained during surgery were excluded. After applying the inclusion criteria, a total of 22 patients from the group with previous bile drainage (Group 1) and 14 patients from the no drainage group (Group 2) were included. The patient selection is shown in Figure 1.

Patients presenting with jaundice or cholangitis underwent biliary drainage via endoscopic retrograde cholangiopancreatography (ERCP) or percutaneous transhepatic cholangiogram (PTC), in accordance with the hospital protocol. All patients received antibiotic prophylaxis with cefuroxime 2 g 30 minutes before skin incision. In all patients, a pancreatoduodenectomy/Whipple procedure was performed, with a Blumgart pancreatojejunostomy, an end-to-side choledochojejunostomy, and a gastrojejunostomy. Two drains were left in place, one near the pancreatojejunostomy and the other near the biliary anastomosis. All patients were submitted to an aseptic bile collection during surgery. Bile culture susceptibility was tested for the most frequently used antibiotics and grouped according to susceptibility patterns. Pancreatic fistulas were categorized according to the International Study Group of Pancreatic Surgery [11].

All data were collected from the electronic files of each patient and analyzed using IBM SPSS Statistics for Windows, Version 28 (Released 2021; IBM Corp., Armonk, New York).

Data collection consisted of demographic data (age and sex), tumor location, associated cholangitis in Group 1, previously used antibiotics, bile cultures and their respective susceptibilities, presence of a pancreatic fistula, and the respective grade.

Continuous variables are expressed as means, while dichotomic data are expressed as frequencies and percentages. All quantitative variables were tested with the Mann-Whitney test when the distribution was not normal and with the t-test when the distribution was normal. The distributions of the continuous variables were evaluated using histograms, Q-Q graphs, and the Shapiro-Wilk test. Age was the only variable that did not follow a normal distribution. Qualitative variables were compared using the chi-square test or Fisher's exact test when the expected cell count was inferior to 5. P-values < 0.05 were considered significant.

## Results

In our cohort, 36 patients underwent pancreatoduodenectomy with the collection of bile samples. Patients' ages ranged from 35 to 84 years, with a mean of 70.72 ± 0.43 years. Most patients were male (22, 61.1%). The main location of the tumor was the pancreas, occurring in 23 (63.9%) of the patients.

Comparing the two groups, the main characteristics of each are described in Table 1. There was no statistical difference between the groups regarding sex, age, and surgical indication, with the most frequent indication being pancreatic adenocarcinoma in both groups.

**Table 1:** Patients' characteristics by group The MW test was used to calculate the p-value of the variable age. The chi-square test was used to calculate the p-values for the variables sex and tumor location. The value of the last column represents the value of the used test. MW: Mann-Whitney U test; X^2^: Chi-square test

Variable	Group 1 (n=22)	Group 2 (n=14)	P-value	Test used
Age (years)	72.4 ± 6.9	68.1 ± 12.2	0.416	MW: 129
Male sex	13 (59.1%)	9 (64.3%)	0.775	X^2^: 0.097
Tumor location				
- Pancreas	14 (63.6%)	9 (64.3%)	0.328	X^2: ^2.229
- Main bile duct	5 (22.7%)	1 (7.1%)		
- Duodenum	3 (13.7%)	4 (28.6%)		

When comparing bile culture results, Group 1 showed a higher positivity rate, with 20 (90.9%) patients having positive bile cultures. Compared with Group 2, only three (21.4%) patients presented with positive bile cultures. Therefore, we found a significant difference in the prevalence of positive bile cultures between the two groups (Table 2).

**Table 2:** Description of positive bile cultures and pancreatic fistulas between the two groups The chi-square test was used to calculate the p-values for the variables sex and tumor location. The value of the last column represents the value of the used test. X^2^: Chi-square

Variable	Group 1 (n=22)	Group 2 (n=14)	P-value	Test used
Positive Bile Cultures	20 (90.9%)	3 (21.4%)	< 0.001	X^2^: 17.902
Pancreatic fistula (any grade)	11 (50%)	3 (21.4%)	0.086	X^2^: 2.939

Microbiology of the bile cultures

In the subgroup of patients that previously received biliary drainage either by ERCP or PTC, only 10 (45.4%) of the patients presented with associated acute cholangitis. Prior to surgery, these patients were treated with broad-spectrum antibiotics. The most commonly used antibiotic in the preoperative setting was piperacillin/tazobactam 4.5 g, used by seven (31.8%) patients.

In Group 1, of the cultures obtained at surgery, only two (9.1%) were negative. Of the remaining 20 (90.9%) positive cultures, 16 (80%) were polymicrobial, and three (15%) also showed fungal growth. There was no statistical difference in the fungal growth regarding the two groups, but polymicrobial cultures were more common in Group 1. When comparing the microbiological susceptibility to the antibiotic used in prophylaxis, cefuroxime, 17 (85%) patients in the group with positive bile cultures presented at least one microorganism that was not susceptible to the standard prophylaxis. These results are presented in Table 3.

**Table 3:** Comparison between the two groups regarding the existence of polymicrobial cultures, fungal growth, and susceptibility to the prophylactic antibiotic used Fisher's exact test was used to calculate the p-values for the three variables presented in the table.

Variable	Group 1 (n=22)	Group 2 (n=14)	P-value
Polymicrobial cultures	16 (80%)	0 (0%)	0.02
Fungal growth	3 (15%)	0 (0%)	0.644
Not susceptible to cefuroxime	17 (85%)	1 (33.3%)	0.107

Table 4 presents the descriptive results of the bile cultures from the two groups.

**Table 4:** Number of positive bile cultures for each microorganism in each group

Bile cultures	Group 1 (n=22)	Total (n)	Group 2 (n=14)	Total (n)
Gram -	K. pneumoniae	9	P. aeruginosa	1
	E. coli	5	E. coli	1
	K. oxytoca	1	-	-
	K. aerogenes	1	-	-
	P. aeruginosa	1	-	-
	Aeromonas caviae	1	-	-
	*E. cloacae *complex	1	-	-
Gram +	E. faecium	9	E. hirae	1
	E. faecalis	3	-	-
	Streptococcus anginosus	4	-	-
	E. avium	2	-	-
	Bifidobacterium	2	-	-
	S. aureus	1	-	-
	Lactobacillus fermentum	1	-	-
	Lactobacillus casei	1	-	-
Fungus	Candida glabrata	1	-	-
	Candida albicans	1	-	-
	Saprochaete clavate	1	-	-
Total		45	-	3

In Group 1, the most frequently isolated Gram-negative bacteria were Klebsiella pneumoniae, and the most frequent Gram-positive bacteria were *Enterococcus faecium. *In this sample, all of the *K. pneumoniae* and *E. coli* bacteria isolated were resistant to cefuroxime. In this group, the antibiotic sensitivities were tested for each of the bacteria and divided according to susceptibility groups; these were divided into bacteria sensitive to first- and second-generation cephalosporins, bacteria only sensitive to piperacillin/tazobactam, only sensitive to carbapenems, only sensitive to ceftazidime/avibactam, only sensitive to vancomycin and to linezolid (Table 5).

**Table 5:** Division of the isolated bacteria according to the susceptibility group

Group of bacteria	Total
Sensitive to first- and second-generation cephalosporins	11 (26.2%)
Sensitive only to piperacillin/tazobactam	16 (38.1%)
Sensitive only to carbapenems	3 (7.1%)
Sensitive only to ceftazidime/avibactam	4 (9.5%)
Sensitive only to vancomycin	7 (16.7%)
Sensitive only to linezolid	1 (2.4%)

Pancreatic fistula

**Figure 2:**
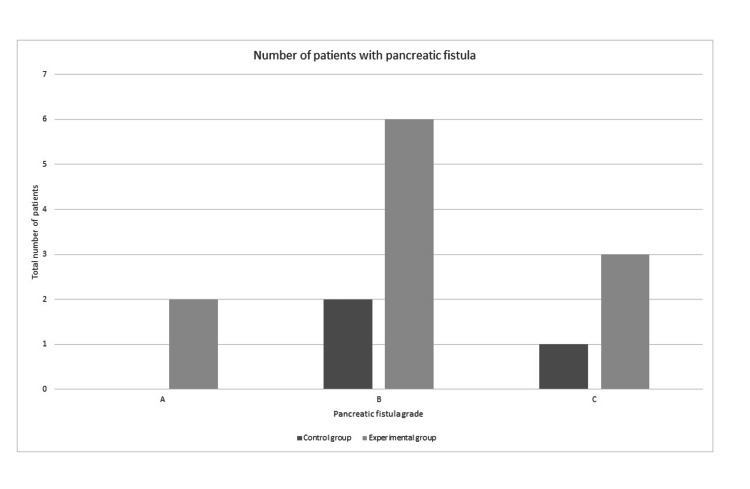
A graphic representation of the fistula grade for each group in the number of patients

Regarding complications, 11 (50%) patients from Group 1 developed a pancreatic fistula in the postoperative period, with three (13.6%) patients having a grade C pancreatic fistula. None of the patients with negative bile cultures presented with POPF. In Group 2, three (21.4%) patients developed a pancreatic fistula. No difference was seen between the two groups regarding pancreatic fistulas, as stated in Table 2. The respective grades are presented in Figure 2. No difference was observed when comparing clinically relevant fistulas between the two groups.

## Discussion

As previously stated, patients with periampullary malignancies often present with jaundice and associated infection and are submitted to preoperative biliary drainage and antibiotic therapy [7]. The most relevant findings of this study are a high percentage of positive bile cultures in patients previously submitted to biliary drainage (90.9%), especially compared with Group 2, and a high percentage of multiresistant microorganisms in bile cultures not covered by standard antibiotic prophylaxis [12].

Biliary drainage alters the normal microbiological profile and increases antibiotic resistance patterns. Positivity in bile cultures for *Enterococcus *spp., *Klebsiella *spp., and *E. coli *was a common finding and was frequently resistant to the common first- and second-generation cephalosporins used in surgical prophylaxis [9,13]. Our study findings are consistent with the previously stated, with *K. pneumoniae* and *E. faecium* being the most frequently isolated microorganisms, and with only 11 (26.2%) of the bacteria being sensitive to the standard surgical prophylaxis used.

A change in the bile microbiologic pattern should lead to a change in antibiotic prophylaxis according to the local pattern of each hospital [14]. The use of broad-spectrum antibiotics as surgical prophylaxis, particularly in patients previously submitted to biliary drainage, leads to a decrease in SSIs and reduced morbidity [14,15]. Broad-spectrum penicillin, such as piperacillin/tazobactam, was the most frequently used antibiotic in clinical trials, with a significant reduction in morbidity and overall mortality when compared with the standard prophylaxis regimen [6,14-16].

Although there has been a shift in the type of antibiotics used for surgical prophylaxis during pancreatoduodenectomy, the optimal duration has not yet been established. Some authors suggest that extending antibiotic treatment to five days could be beneficial [1,17,18].

According to several authors [3-5], bacteriobilia seems to be a risk factor for the development of grade B/C pancreatic fistulas. These authors found that contaminated fistulas were associated with a higher morbidity and mortality, including a higher percentage of postoperative hemorrhage, sepsis, reintervention, and longer hospital stays. Although bacteriobilia does not necessarily correlate with an infected pancreatic fistula, Ohgi et al. [4] concluded that the percentage of positive cultures from the surgical drainage on days 1 and 3 was much more common in patients with positive bile cultures. Nakamura et al. [5] also found that the *E. faecium* species appears to be an independent risk factor for the development of pancreatic fistulas. Although previous studies support an association between bacteriobilia and a higher risk of pancreatic fistulas, this remains controversial, as some studies did not find an increase in pancreatic fistulas in these patients [19]. In our study, the prevalence of clinically relevant pancreatic fistulas (grade B/C) in Group 1 was 9 (40.9%), which is higher than reported in the literature for this patient group. In Group 2, the prevalence of clinically relevant fistulas was 3 (21.4%), but no difference was found between the two groups [3,5].

**Figure 3:**
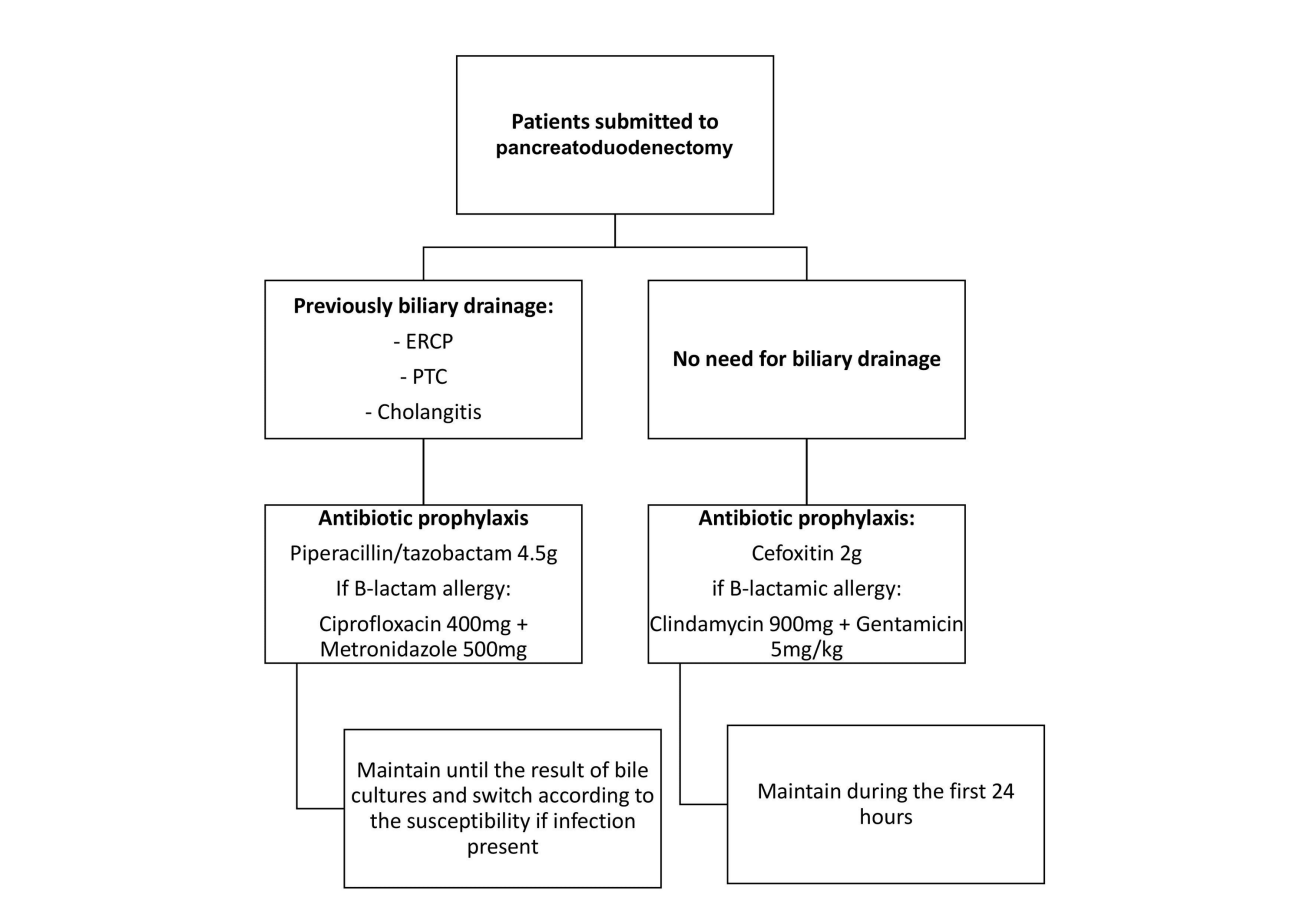
New antibiotic prophylaxis protocol Recent modifications to the prophylactic antibiotic protocol implemented at our institution for patients undergoing pancreatoduodenectomy.

Regarding the current study, we identified a significant shift in microbiological patterns, with a trend toward isolating more multiresistant bacteria. After a multidisciplinary discussion with the infectious diseases team, an adaptation to our antibiotic prophylaxis in patients previously submitted to biliary drainage was made, as seen in Figure 3. To diminish the risk of antibiotic resistance and secondary effects, such as *Clostridium* infections. The antibiotic is discontinued if cultures do not show growth in the first three days, or it is adjusted to the isolated bacteria. This is possible with a close collaboration with the microbiology department. Further plans include analyzing and comparing our data before and after the implementation of the new antibiotic therapy protocol.

Although the presence of positive bile cultures was substantially superior in the drainage group, this did not result in a difference in fistula rates. Some factors that could have contributed to these results were the small sample size and imbalanced distribution between the two groups. There might have been selection bias in the group without drainage, because the criteria for bile collection were not specified, which could have led to a higher prevalence of patients with risk factors for bile contamination. The lack of data comparing the two groups could also lead to a bias regarding pancreatic fistula rates. The microbiological analyses were sufficient to support a change in the antibiotic prophylaxis, but the results regarding the pancreatic fistula rates should be carefully interpreted.

## Conclusions

In conclusion, our study on local microbiological patterns shows a higher prevalence of positive bile cultures in patients previously submitted to biliary drainage. We also found distinct microbial species with varying resistance and susceptibility patterns, rendering the standard prophylactic antibiotic regimen inadequate for these patients. No relationship between the previous instrumentation and pancreatic fistula was found; however, due to the limitations and biases previously stated, this result should be interpreted carefully. This study led to an update of the antibiotic prophylaxis protocol at our institution, and we hope to report further results from implementing these measures.
